# Pressure support ventilation + sigh in acute hypoxemic respiratory failure patients: study protocol for a pilot randomized controlled trial, the PROTECTION trial

**DOI:** 10.1186/s13063-018-2828-8

**Published:** 2018-08-29

**Authors:** Tommaso Mauri, Giuseppe Foti, Carla Fornari, Jean-Michel Constantin, Claude Guerin, Paolo Pelosi, Marco Ranieri, Sara Conti, Daniela Tubiolo, Egle Rondelli, Federica Lovisari, Tommaso Fossali, Savino Spadaro, Domenico Luca Grieco, Paolo Navalesi, Italo Calamai, Tobias Becher, Oriol Roca, Yu-Mei Wang, Rihard Knafelj, Andrea Cortegiani, Jordi Mancebo, Laurent Brochard, Antonio Pesenti, for the Protection Study Group, Giacomo Grasselli, Elena Spinelli, Chiara Abbruzzese, Roberto Rona, Alfio Bronco, Silvia Villa, Stefano Gianni, Alessandra Papoff, Riccardo Pinciroli, Riccardo Colombo, Chiara Sproccati, Pietro Mandelli, Federico Villa, Nicolo’ Patroniti, Iole Brunetti, Lorenzo Ball, Carlo Alberto Volta, Marta Lazzeri, Elisabetta Maragoni, Davide Eleuteri, Giuseppe Bello, Antonio dell’Anna, Eugenio Garofalo, Andrea Bruni, Eugenio Biamonte, Rocco D’Andrea, Lorenzo Querci, Elisabetta Pierucci, Rosario Spina, Irene Mori, Francesco Tomeo, Alain Mercat, François Beloncle, Sebastien Jochmans, Sandie Mazerand, Loredana Baboi, Hodane Yonis, Matthieu Jabaudon, Thomas Godet, Tomas Jovaisa, Tom Barnes, Usman Tariq, Norbert Weiler, Dirk Schädler, Inéz Frerichs, Marina García-de-Acilu, Anxela Vidal, Emilia Rosas, César Pérez Calvo, Jian-Xin Zhou, Spiridon Karagiannis, Vasiliki Zisopoulou, Ioannis Staikos, Marko Noc, Misa Fister, Peter Radsel, Cesare Gregoretti, Ignazio Sabella, Santi Maurizio Raineri

**Affiliations:** 1 Fondazione IRCCS Ca’ Granda Ospedale Maggiore Policlinico, University of Milan, Milan, Italy; 2 ASST Monza, University of Milan-Bicocca, Monza, Italy; 3 Research Centre on Public Health, School of Medicine and Surgery, University of Milan-Bicocca, Monza, Italy; 4 Department of Preoperative Medicine, University Hospital of Clermont-Ferrand, Clermont-Ferrand, France; 5 Service de Réanimation Médicale, Hôpital de la Croix Rousse, Lyon, France; 6 Department of Surgical and Integrated Diagnostics, San Martino Policlinico Hospital, IRCCS for Oncology, University of Genoa, Genoa, Italy; 7 Department of Anesthesia and Intensive Care Medicine, Sapienza University of Rome, Policlinico Umberto I, Rome, Italy; 8 Department of Anesthesia and Critical Care, Niguarda Hospital, University of Milan-Bicocca, Milan, Italy; 9 Department of Anesthesiology and Intensive Care, ASST Fatebenefratelli Sacco - Luigi Sacco Hospital, Milan, Italy; 10 Department of morphology, surgery and experimental medicine, Azienda Ospedaliera-Universitaria Arcispedale Sant’Anna, Ferrara, Italy; 11 Department of Anesthesiology and Intensive Care Medicine, Catholic University of The Sacred Heart, IRCCS Fondazione Policlinico A. Gemelli, Rome, Italy; 12 Dipartimento di Scienze Mediche e Chirurgiche, Università Magna Graecia di Catanzaro, Azienda Ospedaliera Universitaria Mater Domini, Catanzaro, Italy; 13 AUSL Toscana Centro, Unit of Anesthesia and Resuscitation, San Giuseppe Hospital, Empoli, Italy; 14 Department of Anesthesiology and Intensive Care Medicine, University Medical Center Schleswig-Holstein, Campus Kiel, Kiel, Germany; 15 Critical Care Department, Vall d’Hebron University Hospital, Vall d’Hebron Research Institute, Universitat Autònoma de Barcelona, Barcelona, Spain; 16 Department of Critical Care Medicine, Beijing Tiantan Hospital, Capital Medical University, Beijing, China; 17 Center for Internal Intensive medicine (MICU), University Medical Center Ljubljana, Ljubljana, Slovenia; 18 Department of Biopathology and Medical Biotechnologies (DIBIMED), Section of Anesthesia, Analgesia, Intensive Care and Emergency, Policlinico Paolo Giaccone, University of Palermo, Palermo, Italy; 19 Servei de Medicina Intensiva, Hospital de la Santa Creu i Sant Pau, Universitat Autònoma de Barcelona (UAB), Barcelona, Spain; 20 Interdepartmental Division of Critical Care Medicine, University of Toronto, Toronto, ON Canada; 21 Keenan Research Centre, Li Ka Shing Knowledge Institute, St. Michael’s Hospital, Toronto, ON Canada; 22 Istituto Clinico Humanitas, Milan, Italy; 23 Department of Anesthesiology, Intensive Care and Transplants, University Hospital St. Orsola-Malpighi, Bologna, Italy; 24 Département de Médecine Intensive - Réanimation, CHU d’Angers, Université d’Angers, Angers, France; 25 Department of Intensive Care Medicine, Sud Ile-de-France Hospitals, Melun, France; 26 BHR University Hospitals NHS Trust, Romford, Greater London UK; 27 Hospital Universitario Fundación Jiménez Díaz de Madrid, Madrid, Spain; 28 Intensive Care Unit, Larissa General Hospital, Larissa, Greece; 29 Anesthesia and Critical Care, Department of Pathophysiology and Transplantation, University of Milan, Milan, Italy; 30 Department of Anesthesia, Critical Care and Emergency, Fondazione IRCCS Ca’ Granda Ospedale Maggiore Policlinico, Via F. Sforza 35, 20122 Milan, Italy

**Keywords:** Mechanical ventilation, Intervention study, Ventilator-induced lung injury, Positive-pressure ventilation, Sigh, Recruitment, Weaning, Pressure support

## Abstract

**Background:**

Adding cyclic short sustained inflations (sigh) to assisted ventilation yields optimizes lung recruitment, decreases heterogeneity and reduces inspiratory effort in patients with acute hypoxemic respiratory failure (AHRF). These findings suggest that adding sigh to pressure support ventilation (PSV) might decrease the risk of lung injury, shorten weaning and improve clinical outcomes. Thus, we conceived a pilot trial to test the feasibility of adding sigh to PSV (the PROTECTION study).

**Methods:**

PROTECTION is an international randomized controlled trial that will be conducted in 23 intensive care units (ICUs). Patients with AHRF who have been intubated from 24 h to 7 days and undergoing PSV from 4 to 24 h will be enrolled. All patients will first undergo a 30-min sigh test by adding sigh to clinical PSV for 30 min to identify early oxygenation responders. Then, patients will be randomized to PSV or PSV + sigh until extubation, ICU discharge, death or day 28. Sigh will be delivered as a 3-s pressure control breath delivered once per minute at 30 cmH_2_O. Standardized protocols will guide ventilation settings, switch back to controlled ventilation, use of rescue treatments, performance of spontaneous breathing trial, extubation and reintubation. The primary endpoint of the study will be to verify the feasibility of PSV + sigh evaluated through reduction of failure to remain on assisted ventilation during the first 28 days in the PSV + sigh group versus standard PSV (15 vs. 22%). Failure will be defined by switch back to controlled ventilation for more than 24 h or use of rescue treatments or reintubation within 48 h from elective extubation. Setting the power to 80% and first-risk order to 5%, the computed size of the trial is 129 patients per arm.

**Discussion:**

PROTECTION is a pilot randomized controlled trial testing the feasibility of adding sigh to PSV. If positive, it will provide physicians with an effective addition to standard PSV for lung protection, able to reduce failure of assisted ventilation. PROTECTION will provide the basis for a future larger trial aimed at verifying the impact of PSV + sigh on 28-day survival and ventilator-free days.

**Trial registration:**

ClinicalTrials.gov, NCT03201263. Registered on 28 June 2017.

**Electronic supplementary material:**

The online version of this article (10.1186/s13063-018-2828-8) contains supplementary material, which is available to authorized users.

## Background

Although mechanical ventilation is an effective support strategy for patients with acute hypoxic respiratory failure (AHRF), it has the potential to aggravate lung damage through the development of ventilation-induced lung injury (VILI) [1]. Ever since a landmark study demonstrated the association between the use of high tidal volumes during controlled mechanical ventilation and increased mortality [2], “protective” controlled ventilation with limited volume and pressure has become the recommended treatment for patients with AHRF, in order to minimize the risk of VILI [3]. Volutrauma and barotrauma (i.e., the key mechanisms causing VILI during controlled ventilation) might also occur during assisted ventilation, if excessive force generated by the respiratory muscles adds to the pressure delivered by the ventilator to determine high tidal volume and injurious transpulmonary pressure [4]. However, while protective strategies have been established for patients undergoing controlled mechanical ventilation (e.g., limiting plateau pressure to < 30 cmH_2_O), interventions that enhance lung protection during assisted ventilation are still lacking [5]. Around 30% of invasively ventilated patients with AHRF already breath spontaneously by day 1 after intubation [6] and early implementation of specific strategies to enhance lung protection during assisted ventilation may have a tremendous impact on clinical practice.

From a pathophysiological standpoint, during assisted ventilation, derecruitment and lung heterogenity may facilitate the generation of high regional barotrauma and volutrauma [7], thus increasing the risk of VILI [8–10]. The addition of cyclic short sustained inflations (sigh) to assisted ventilation is known to optimize recruitment and decrease heterogeneity in patients with AHRF [11–16]. Moreover, sigh might reduce the inspiratory effort and the tidal volume [11–16]. These physiologic studies generated the hypothesis that addition of sigh to pressure support ventilation (PSV, the most common assisted mechanical ventilation mode) might decrease the risk of VILI, potentially yielding faster weaning and improved clinical outcomes.

As prospective clinical trials including long-term use of sigh during assisted ventilation have not been performed yet, we conceived a pilot randomized controlled trial (RCT) to verify the clinical feasibility of the addition of sigh to PSV (PSV + sigh) in comparison to standard PSV. Therefore, the objective of the present trial is to determine whether ventilation by PSV + sigh can be applied early and safely in intubated patients with AHRF and successfully maintained until extubation. Specifically, the primary endpoint is to evaluate whether PSV + sigh, as compared to PSV, is associated with reduced failure of assisted ventilation, with failure defined as (1) prolonged switch to controlled ventilation; (2) use of rescue therapies for refractory hypoxia or (3) early need for reintubation after elective extubation.

## Methods

### Design

**Fig. 1:**
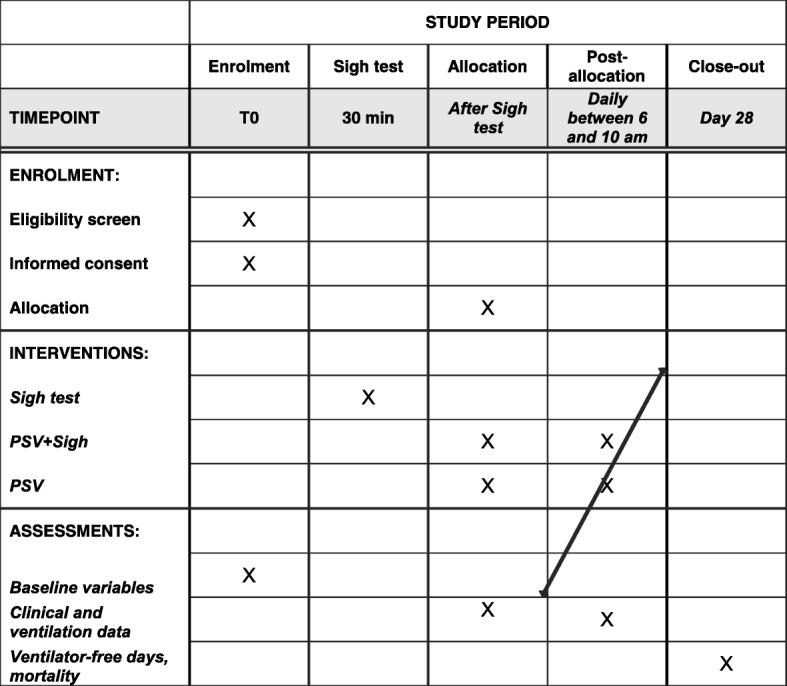
Standard protocol items: recommendation for interventional trials (SPIRIT) figure. Schedule of enrolment, interventions, and assessments for the PROTECTION trial. PSV, pressure support ventilation

The PROTECTION (Pressure support ventilation + sigh in acute hypoxemic respiratory failure patients) trial is an investigator-initiated, international, multicenter, parallel randomized controlled two-arm trial that will be performed in intubated and ventilated patients with AHRF, who are admitted to the intensive care unit (ICU). The study will be conducted in adherence to the principles of the World Medical Association’s Declaration of Helsinki and in accordance with the Medical Research Involving Human Subjects Act (WMO). Ethics approval will be sought from each participating institution before starting enrollment and consent will be obtained for each patient following local regulations. The Institutional Review Board of the coordinating center approved the protcol on 12 June 2017 under reference number 318_2017bis. The trial was registered at www.clinicaltrials.gov with code NCT03201263 in June 2017. The Standard protocol items: recommendation for interventional trials (SPIRIT) checklist can be found in Additional file 1 and the SPIRIT figure is included in the main body of the manuscript (Fig. 1).

### Setting

The PROTECTION trial will be performed in the ICUs of 23 centers around the world (12 in Italy, 10 in other European countries, 1 in China). The coordinating center will be Policlinico Maggiore Hospital in Milan, Italy.

### Study population

Consecutive patients admitted with AHRF to each participating ICU, who have been intubated and switched to PSV between 1 and 7 days after intubation will be screened between 4 and 24 h from the start of PSV. Further inclusion criteria will be partial arterial pressure of oxygen (PaO_2_)/fraction of inspired oxygen (FiO_2_) ratio ≤ 300 mmHg (measured at clinical positive end-expiratory pressure (PEEP) and FiO_2_ values); clinical PEEP ≥ 5 cmH_2_O and stable Richmond Agitation-Sedation Scale (RASS) value of − 2 to 0. Exclusion criteria will be clinical PEEP ≥ 15 cmH_2_O; partial pressure of arterial carbon dioxide (PaCO_2_) > 60 mmHg; arterial pH < 7.30; PaO_2_/FiO_2_ ratio ≤ 100 mmHg (measured at clinical PEEP and FiO_2_ values); age < 18 years; presence of central nervous system or neuromuscular disorders; history of severe chronic obstructive pulmonary disease or fibrosis; AHRF fully explained by cardiac failure or fluid overload; evidence of active air leak from the lung (e.g., pneumothorax); cardiovascular instability (e.g., systolic blood pressure (SBP) < 90 mmHg despite vasopressors); clinical suspicion of elevated intracranial pressure; extracorporeal support; moribund status and refusal by the attending physician.

If an eligible patient is excluded from participation, the reason(s) for exclusion will be registered.

**Fig. 2:**
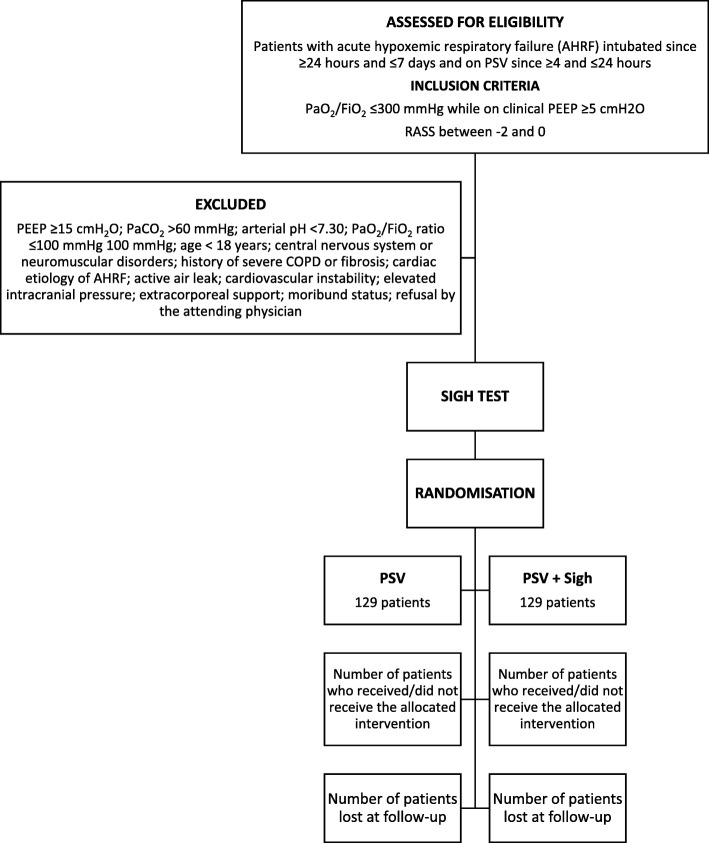
The Consolidated Standards of Reporting Trials (CONSORT) diagram of the PROTECTION trial. PaO_2_, partial pressure of arterial oxygen; FiO_2_, fraction of inspired oxygen; PEEP, positive end-expiratory pressure; RASS, Richmond Agitation-Sedation Scale; PaCO_2_, partial pressure of arterial carbon dioxide; COPD, chronic obstructive pulmonary disease; PSV, pressure support ventilation

The Consolidated Standards of Reporting Trials (CONSORT) diagram of PROTECTION trial is presented in Fig. 2.

### Sigh test

To explore the possibility of implementing predictive enrichment in a larger future RCT [17], we planned a pre-randomization test to assess the prevalence of patients with AHRF with improved oxygenation after the introduction of sigh. After enrollment, FiO_2_ will be titrated to obtain pulse arterial oxygen saturation (SpO_2_) of 90–96% maintaining clinical PSV settings including PEEP; then, all patients will undergo a clinical test of PSV + sigh to assess the prevalence of improved oxygenation in sigh responders vs. non-responders. To this end, we will first record the SpO_2_/FiO_2_ ratio at the start of the test; then, 30 min after the introduction of sigh as the cyclic pressure control phase set at 30 cmH_2_O for 3 s once per minute [14], SpO_2_/FiO_2_ will be collected again to quantify the number of patients in whom this ratio increased (i.e., “sigh responders”) [18]. To deliver sigh, ventilators will be switched to biphasic positive airway pressure mode with the lower pressure-level set at clinical PEEP and the higher pressure-level of the sigh set at 30 cmH_2_O with a 3-s inspiratory time and then a 57-s expiratory time [16].

### Randomization

At the end of the sigh test, patients will be randomized through an online automatic centralized and computerized system to one of the two study groups (1:1 ratio): PSV or PSV + sigh. Due to the nature of the intervention, blinding will not be feasible.

### Interventions: ventilation strategies for each study arm

#### PSV group

After randomization, clinicians will set PSV to meet the following targets: tidal volume (Vt) of 6–8 mL/kg of predicted body weight (PBW), with respiratory rate (RR) 20–35 bpm. FiO_2_ will be left as selected before the pre-randomization sigh test, while PEEP will be left as clinically set.

#### PSV + sigh group

PSV will be set with the same protective targets of the PSV group (see above) but cyclic pressure control phase at 30 cmH_2_O for 3 s delivered once per minute (i.e., sigh) [14] will be added. FiO_2_ will be left as selected before the pre-randomization sigh test with clinical PEEP.

#### Adjusting ventilation settings

In both groups, PSV will be adjusted, at least every 8 h, as follows:

- PSV support will be decreased by 2 cmH_2_O if Vt > 8 mL/kg PBW and/or RR < 20
- PSV support will be increased by 2 cmH_2_O if Vt < 6 mL/kg PBW and/or RR > 35 and/or in the presence of respiratory distress (e.g., marked use of the accessory muscles)
- PEEP and then FiO_2_ will be increased by 2 cmH_2_O and 0.1 if SpO_2_ is < 90%
- FiO_2_ and then PEEP will be decreased by 0.1 and 2 cmH_2_O if SpO_2_ is > 96%.

The PSV group will be treated by protective PSV settings until day 28 or death or performance of a spontaneous breathing trial (SBT); the PSV + sigh group will be treated by protective PSV settings with the addition of sigh until day 28 or death or performance of a spontaneous breathing trial (SBT). Sigh settings will be left unchanged until day 28, death or the SBT.

#### Switch to controlled mechanical ventilation

In both groups, switch to protective controlled ventilation will be indicated if patient develops at least one of the following conditions: PSV support > 20 cmH_2_O; PEEP ≥ 15 cmH_2_O; unstable hemodynamic status (SBP < 90 mmHg with vasoactive drug); active cardiac ischemia (dynamic ST changes on cardiac monitor or electrocardiogram); unstable arrhythmias; uncontrolled hypertension (SBP > 180 mmHg); abrupt decrease in the level of consciousness (RASS <− 3); dangerous agitation (RASS > + 2); pH < 7.30; PaO_2_/FiO_2_ ratio ≤ 100 mmHg; necessity to perform diagnostic test (e.g., computed tomography (CT scan) or bronchoscopy).

Controlled ventilation will be set on volume mode with Vt 6–8 mL/kg PBW, RR to control pH, unchanged PEEP and FiO_2_. Controlled ventilation will be thereafter adjusted according to clinical evolution. Patients switched to controlled ventilation will be reassessed at least every 8 h and they will be switched back to PSV or PSV + sigh (to maintain study group assignment) targeting the aforementioned settings and adjustments as soon as all the following conditions are met: patient is able to trigger ventilator breaths; PaO_2_/FiO_2_ > 100 mmHg; PEEP < 15 cmH_2_O; pH ≥ 7.3; stable hemodynamic status with stable or decreasing doses of vasopressors for ≥ 6 h.

#### Rescue therapies

In the case of desaturation (SpO_2_ ≤ 90%) it will be crucial to exclude hemodynamic impairment as a possible cause. Also, airway obstruction and ventilator malfunction must be ruled out. Provided those factors are excluded, a rescue step-up strategy will be allowed: institution of protective controlled mechanical ventilation (see above for settings) and performance of recruitment maneuvers at 40–50 cmH_2_O, PEEP ≥ 15 cmH_2_O, prone positioning and inhaled nitric oxide, extracorporeal membrane oxygenation. Patients undergoing controlled ventilation and rescue treatments will be reassessed at least every 8 h and switched back to PSV or PSV + sigh (to maintain study group assignment) with the aforementioned settings and adjustments as soon as all the following conditions are met: patient can trigger ventilator breaths; PaO_2_/FiO_2_ > 100 mmHg; PEEP < 15 cmH_2_O; pH ≥ 7.3 and stable hemodynamic status with stable or decreasing doses of vasopressors for ≥ 6 h.

### Weaning protocol and criteria for reintubation

#### SBT

Patients with SpO_2_ ≥ 90% on FiO_2_ ≤ 0.4 and PEEP ≤ 5 cmH_2_O, no agitation, hemodynamically stable with norepinephrine ≤ 0.1 μg/kg/min or equivalent and at a stable or decreasing dose for 6 h [19] and without any of the aforementioned criteria for switch to controlled ventilation will undergo the SBT. For patients in the PSV group, the attending physician will perform the SBT directly. For patients in the PSV + sigh group, the attending physician will first withdraw sigh, wait 60 min and confirm the criteria: if confirmed, the SBT will be performed; if not, sigh will be reintroduced and the clinical criteria will be checked again to repeat the procedure after at least 8 h.

The SBT will last at least 60 min with a combination of PEEP 0–5 cm H_2_O and PSV 0–5 cm H_2_O. At the end of the 60 min, the patient will fail the SBT if any of the following are present: criteria to start the SBT not confirmed; sustained (> 5 min) respiratory rate > 35 bpm; heart rate > 140 bpm; SBP > 180 or < 80 mmHg; marked complaint of dyspnea; increased somnolence with elevated PaCO_2_ and/or pH < 7.3; active cardiac ischemia (dynamic ST changes on cardiac monitor or electrocardiogram); abrupt decrease in the level of consciousness with RASS <− 3 or if cough is not strong enough to clear secretions.

Patients who fail the SBT will be switched back to PSV or PSV + sigh (to maintain study group assignment) and clinical criteria will be checked again to repeat the procedure after at least 6 h.

Patients who pass the SBT will be extubated or, in the presence of tracheostomy, mechanical ventilation will be discontinued. If a patient is reintubated or mechanically ventilated through a tracheostomy again within 48 h, PSV or PSV + sigh (to maintain study group assignment) will be restored. If a patient remains extubated or separated from the ventilator for > 48 h data collection only will continue.

#### Reasons for reintubation

After elective extubation, reintubation should be promptly performed if at least one of the following criteria is present: cardiac arrest; respiratory arrest (respiratory pauses with loss of consciousness or gasping for air); respiratory failure with SpO_2_ < 90% and/or RR > 35 bpm despite non-invasive ventilation; decreased level of consciousness impairing ability to protect airway; hemoptysis or hematemesis impairing ability to protect airway; abundant secretions that cannot be effectively cleared or are associated with lobar collapse, acidosis, hypoxemia or change in mental status; surgical/invasive procedure requiring sedation/anesthesia +/− neuromuscular blockade such that patient is no longer be able to sustain unassisted breathing or hemodynamic instability with SBP < 80 mmHg despite vasoactive drugs.

### Standard of care

In all patients, standard of care for intubated patients with hypoxemic acute respiratory failure (e.g., restrictive fluid strategy, early appropriate antibiotics, prophylaxis of gastric stress ulcer and deep vein thrombosis, semi-recumbent positioning, respiratory physiotherapy, adequate nutrition, monitoring of sedation, pain and delirium, tracheostomy and non-invasive ventilation for post-extubation respiratory failure) will be granted throughout the whole ICU stay in accordance to local protocols.

### End of follow up

Enrolled patients will be observed until day 28, ICU discharge or death, whichever comes first.

### Study endpoints

#### Primary endpoint

The primary endpoint will be to verify the clinical feasibility of PSV + sigh vs. PSV. Feasibility will be assessed by comparing the number of patients in each group experiencing at least one of the following failure criteria [20]: switch to controlled ventilation following presence of one of the aforementioned reasons for ≥ 24 h consecutively; use of PEEP ≥ 15 cmH_2_O, prone positioning, inhaled nitric oxide, extracorporeal membrane oxygenation or reintubation within 48 h from extubation following one of the aforementioned reasons.

#### Secondary endpoints

The study will also provide a preliminary evaluation of the safety of PSV + sigh by comparing the incidence of the following adverse events in the two study groups: hemodynamic instability with hypotension (i.e., SBP < 90 mmHg) despite vasoactive drugs; arrhythmias with heart rate < 40 or > 140 bpm; radiographic evidence of barotrauma (i.e., pneumothorax, pneumomediastinum, pneumatocoele or subcutaneous emphysema or new chest tube placement.

Furthermore, it will quantify the prevalence of short-term (i.e., within 30 min) and long-term (i.e., within 24 h in the PSV + sigh group) sigh responders defined by improved oxygenation. Finally, it will assess the following clinical outcomes: 28-day mortality; ventilator-free days; PSV level, PEEP value and oxygenation index on day 1–3; number of days on assisted ventilation until day 28; use of rescue treatments; number of quadrants involved on chest x-ray on day 1–5; ICU and hospital length of stay and tracheostomies. The listed variables will be analyzed by comparing the two study groups considering, first, the whole study population and, then, only patients who improved oxygenation during the pre-randomization sigh test (sigh responders).

### Data collection

At enrollment, before the sigh test we will anonymously collect patients’ demographic information (e.g., age, sex, height, weight), past (e.g., hypertension, chronic medications) and recent (e.g., etiology of the acute respiratory failure, days since intubation) medical history, severity of lung injury (e.g., ventilation setting, arterial blood gases, respiratory system compliance, diagnosis of acute respiratory distress syndrome (ARDS)) and of systemic involvement (e.g., presence of shock, number of organ failures) and finally, ventilation data (e.g., RR, Vt, PEEP, FiO_2_, PSV level).

In both groups, for the first 24 h, we will assess the SpO_2_/FiO_2_ ratio, RR and PSV tidal volume every 4 h to further characterize physiologic response to sigh over time.

From day 1 (i.e., within 24 h from randomization) to day 28 or death or discharge from the ICU, the following data will be collected every day between 6:00 and 10:00 in the morning: arterial SpO_2_, arterial and central venous blood gas analyses, ventilation settings and pattern (i.e., sigh pressure level, sigh tidal volume, PSV level, PSV tidal volume, respiratory rate, PEEP, FiO_2_, minute ventilation, P0.1, mean airway pressure), heart rate, arterial blood pressure, central venous pressure, cumulative fluid balance, patient’s comfort assessed using a visual analog scale (VAS), RASS value, SOFA score and dosage of sedative agents and vaso-active drugs.

Moreover, each day we will collect information on use of rescue treatments (i.e., recruitment manoeuvers, use of PEEP ≥ 15 cmH_2_O, prone positioning, inhaled nitric oxide, extracorporeal membrane oxygenation), tracheostomy, switch from the allocated treatment to the other study arm for ≥ 24 h, switch to controlled ventilation for ≥ 24 h, reason for switch to controlled ventilation, adverse events (i.e., hemodynamic instability with hypotension with SBP < 90 mmHg despite vasoactive drugs; arrhythmias; radiographic evidence of barotrauma with pneumothorax, pneumomediastinum, pneumatocoele, or subcutaneous emphysema), SBT performance and reason for SBT failure, extubation or separation from mechanical ventilation, reintubation and reason for reintubation with time. We will collect data on day 28 for all enrolled patients on mortality, extubation that occurred outside the ICU and readmission to ICU.

### Statistical considerations

Statistical support for the PROTECTION trial will be granted by collaboration with Dr Carla Fornari and Dr Sara Conti from the Research Center on Public Health, Department of Medicine and Surgery, University of Milano-Bicocca, Monza, Italy. Drs Fornari and Conti are PhD statisticians with experience in public health and physiologic studies and they participated to PROTECTION study conception, draft of the study protocol, performed the power analysis to decide sample size and prepared the data analysis plan.

### Sample size

The sample size was computed based on the hypothesis that PSV + sigh might decrease the rate of failure of assisted ventilation compared to standard PSV. Based on previous data [21], the expected proportion of failure in patients undergoing PSV will be 22% and we hypothesized a proportion of 15% for patients in the PSV + sigh group. Assuming non-inferiority of PSV + sigh treatment, with a tolerance of 5% we estimated that a sample size of 258 patients (with 129 patients per study arm) will be sufficient to assess feasibility of the PSV + sigh strategy in this pilot phase with power of 0.8 and alpha 0.05.

### Statistical analysis

Continuous normally distributed variables will be expressed by their mean and standard deviation or as medians and their interquartile ranges when not normally distributed. Categorical variables will be expressed as number and proportion (%). To assess the feasibility of PSV + sigh as compared to PSV (i.e., the primary endpoint), we will compute the proportion of patients experiencing at least one of the following events in each arm: switch to controlled ventilation for ≥ 24 h consecutively; use of PEEP ≥ 15 cmH2O, prone positioning, inhaled nitric oxide, extracorporeal membrane oxygenation or reintubation within 48 h from extubation.

We will compare the two proportions using the one-tailed non-inferiority test for proportions with a 5% tolerance. We will use 0.05 as the significance level. The same method will be adopted to evaluate the safety of PSV + sigh, which is a secondary endpoint. Prevalence of sigh responders in the two arms will be compared using the chi-squared or Fisher’s exact test. Ventilator-free days will be calculated as the difference between the duration of follow up (up to 28 days) and the number of days of intubation before successful extubation or separation from mechanical ventilation for tracheostomized patients. For all the secondary endpoints, we will use the chi-squared or Fisher’s exact test in the case of binary variables or the *t* test or Wilcoxon signed rank test for continuous variables. Time to death or extubation will be analyzed using Kaplan-Meier survival curves. Adjustement for potential confounding factors will be evaluated using appropriated regression models.

### Study organization

The steering committee is composed of two principal investigators (TM and LB) plus seven international experts on ventilation who contributed to the design and revision of the study protocol. Each participating center will indicate a local investigator in charge of the study. The principal investigators are responsible for administrative management and communication with the local investigators and for helping the participating clinical sites in trial management, record keeping and data management. The local investigators provide structural and scientific leadership. They guarantee the integrity of data collection and ensure timely completion of the case report forms.

Collected data will be entered in an electronic case report form (eCRF) available online at a dedicated website (https://sighprotection.digitalcrf.eu), with protected individual access for each participating center. Patient data will be anonymous and coded according to a number. The eCRF includes tools to promote data quality, such as range checks for data values. Data monitoring will be performed by means of queries on the database done by statisticians and analyzed to identify abnormalities and inconsistencies.

Based on clinical experience and results of physiologic studies, the additional risks for patients enrolled in the PSV + sigh arm is expected to be minimal in comparison to standard of care. Still, specific patient insurance will be granted to cover all unexpected adverse events caused by the study interventions and all adverse events will be monitored by and reported to the coordinating center.

## Discussion

PROTECTION is a pilot randomized controlled trial to test the long-term clinical feasibility of the addition of sigh to PSV in comparison to standard PSV. If positive, it will provide the basis for a future larger trial aimed at verifying the impact of PSV + sigh on a composite outcome endpoint including 28-day survival and ventilator-free days. Moreover, it will provide physicians a physiologically sound addition (i.e., sigh) to standard PSV for lung protection able to favor assisted ventilation.

Mortality remains considerably high (around 40%) in intubated patients with AHRF [6]. The clinical severity of lung injury at presentation and early appropriate treatment are the main determinants of patients’ outcomes [19]. However, while the lungs recover, VILI might significantly impact survival [1]. Recent studies testing strategies that enhance lung protection (e.g., early referral to an extracorporeal membrane oxygenation (ECMO) center [22]; use of muscle relaxants during the first 48 h after intubation [23]; early use of extended sessions of prone positioning [24]) yielded significantly decreased mortality. However, such trials enrolled only the most severely ill patients with AHRF on controlled mechanical ventilation. A recent large observational study [6], instead, showed that most ventilated patients admitted to the ICU have mild to moderate AHRF or ARDS that leads to considerably high mortality (≈ 35%) anyway. Moreover, the same study reported that around 30% of invasively ventilated patients with AHRF and ARDS are on some form of spontaneous breathing from day 1 after intubation, independent of the severity of their illness [6], and this proportion likely increases within 1 week. Thus, there is an urgent need for interventions that increase lung protection during assisted ventilation.

Key determinants of VILI in patients with AHRF undergoing assisted ventilation include elevated ventilation pressures and inspired fraction of O_2_ (FiO_2_) [25]; heterogeneous distribution of alveolar collapse that increases the regional tidal volume/end expiratory lung volume (Vt/EELV) ratio (i.e., the regional lung strain) [8] and strenuous inspiratory effort and low regional compliance that determines elevated regional transpulmonary pressure (i.e., the regional lung stress) [10]. To this end, previous studies showed that the addition of sigh to assisted mechanical ventilation improves oxygenation without increasing ventilation pressures and FiO_2_; decreases the tidal volumes by decreasing the patient’s inspiratory drive; increases the EELV by regional alveolar recruitment; decreases regional heterogeneity of lung parenchyma; decreases patients’ inspiratory efforts limiting transpulmonary pressure and improves regional compliance [11–16, 26]. Of note, the improvement in gas exchange and lung mechanics disappears after sigh discontinuation [12]. In conceiving this study, we reasoned that long-term application of sigh during PSV might decrease the risk of VILI through various synergic mechanisms, possibly decreasing the duration of mechanical ventilation and mortality in patients with AHRF. To date, no RCT has tested the long-term safety and feasibility of the application of sigh. Therefore, before testing the effects of sigh on clinical outcome in a RCT, the feasibility of long-term use of sigh had to be investigated: this is the primary aim of the PROTECTION trial. Moreover, data on the safety of sigh will be collected, evaluating potential adverse effects including development of pneumothorax or other signs of barotrauma and hemodynamic instability. Finally, the sample size was calculated based on a potential deacrese in failure to remain on assisted ventilation from 22% during PSV to 15% during PSV + sigh, so that the potential to promote spontaneous breathing by addition of sigh will already be tested.

We anticipate the PROTECTION trial to be highly feasible, as study procedures are mainly limited to ventilation settings and do not include complex interventions, with limited burden on daily activities. PSV + sigh is an easy-to-implement ventilation mode and, for the present study, we will use high-performance ICU ventilators already available in each clinical unit, dramatically decreasing the study costs.

Even though PROTECTION might be a largely pragmatic study, the protocol specifically suggests adjustments of ventilation settings and weaning procedures. Moreover, criteria for switch to controlled ventilation, use of rescue therapies and reintubation are pre-defined by the study protocol. Since return to controlled mechanical ventilation can occur due to reasons not related to failure of assisted ventilation (e.g., necessity to perform diagnostic or therapeutic procedures requiring deep sedation and neuromuscular blockade), the protocol stresses the importance of periodic reassessment (at least every 8 h) to restore assisted ventilation as soon as clinical conditions permit. To this end, failure of assisted ventilation is defined when controlled ventilation is maintained for at least 24 h. In summary, the PROTECTION study protocol limits discretional settings and clinical decisions that might interfere with the primary outcome.

One important limitation of the PROTECTION trial is that blinding is not possible due to the nature of the intervention, and this could induce bias. However, all the processes that can influence the primary outcome are regulated by the protocol and all the analyses will be performed in a blinded fashion. Second, even though a lower sigh rate might be regarded as more physiological [15], in the absence of conclusive data on sigh “minimum effective dose” [14], we chose a rate of one per minute because this can be performed by all the clinically available high-performance ICU ventilators, increasing feasibility and decreasing costs, especially of the future larger RCT. Finally, we did not plan any biomolecular analysis to verify the impact of addition of sigh on lung protection for budget reasons; in any case, we will collect a huge amount of physiological data during the whole study period that will allow reconstruction on mechanistic effects underlying the clinical benefits.

In conclusion, PROTECTION is a pilot randomized controlled trial to test the feasibility of long-term addition of sigh to PSV. Its results could provide a ready-to-use treatment enhancing successful application of assisted ventilation. Moreover, PROTECTION will be the basis for planning a future larger trial investigating the use of sigh as a strategy to improve hard clinical outcomes in patients with AHRF undergoing assisted ventilation through enhanced lung protection.

## Trial status

The PROTECTION trial is currently recruiting patients. The protocol was approved by the coordinating center Institutional Review Board on 12 June 2017. The first patient was enrolled on 20 December 2017. Sixteen centers already received approval from the local ethical committees and are actively recruiting patients. Recruitment is expected to be completed in November 2019.

## Additional file

Additional file 1: SPIRIT 2013 checklist: Recommended items to address in a clinical trial protocol and related documents. (DOC 121 kb)

## Abbreviations

AHRF: Acute hypoxemic respiratory failure
ARDS: Acute respiratory distress syndrome
CONSORT: Consolidated Standards of Reporting Trials
ECMO: Extra-corporeal membrane oxygenation
eCRF: Electronic case report form
EELV: End-expiratory lung volume
ESICM: European Society of Intensive Care Medicine
FiO_2_: Fraction of inspired oxygen
ICU: Intensive care unit
PaCO_2_: Partial arterial pressure of carbon dioxide
PaO_2_: Partial arterial pressure of oxygen
PBW: Predicted body weight
PEEP: Positive end-expiratory pressure
PSV: Pressure support ventilation
RASS: Richmond Agitation-Sedation Scale
RCT: Randomized controlled trial
RR: Respiratory rate
SBP: Systolic blood pressure
SBT: Spontaneous breathing trial
SOFA: Sequential Organ Failure Assessment
SPIRIT: Standard protocol items: recommendation for interventional trials
SpO_2_: Pulse arterial oxygen saturation
VILI: Ventilator-induced lung injury
Vt: Tidal volume
